# Supplementary material for: The genome sequence of a lauxaniid fly, Tricholauxania praeusta (Fallén, 1820)
Source: Wellcome Open Res. 2024 Oct 15;9:586. [Version 1] doi: 10.12688/wellcomeopenres.23099.1 (PMC11574340; doi:10.12688/wellcomeopenres.23099.1)
Supplement: Peer review history

# Reviewer report: Reviewer response for version 1

**DOI:** 10.21956/wellcomeopenres.25436.r109627

**Published:** 2024-11-18

**Authors:** Alexis L Sperling

**Affiliations:** 1 University of Cambridge, Cambridge, England, UK

This is a report detailing the information for the genome sequence and assembly of *Tricholauxania praeusta. *I would have enjoyed reading a little more about the biology of this fly, if much is known, but sufficient information was given. 

 The data presented in Tables 1/2 and Figure 2 show that the genome assembly is exceptionally good. They have produced a true chromosome-level genome assembly that is effectively phased. The BUSCO score is good but the number of transcripts is close to that of the number of predicted genes. The BlobToolKit data shows that there is effectively no genome sequence contamination (Figures 3/4). The Hi-C contact map (Figure 5) shows a very good and confident assembly, matching the rest of the metrics presented in the paper. The data is very good, the GC content is uniform across the genome (Table 3). It has the classic equivalent to 5 chromosome arms and the dot chromosome, like other dipterans. It would be easy to identify the sex chromosome by comparing the sequence to *D. melanogaster*, it is likely chromosome 2.

 This is an exceptionally good genome assembly and very useful report detailing all of the relevant information.

Are sufficient details of methods and materials provided to allow replication by others?

Yes

Is the rationale for creating the dataset(s) clearly described?

Yes

Are the datasets clearly presented in a useable and accessible format?

Yes

Are the protocols appropriate and is the work technically sound?

Yes

Reviewer Expertise:

Dipteran parthenogenesis

I confirm that I have read this submission and believe that I have an appropriate level of expertise to confirm that it is of an acceptable scientific standard.

# Reviewer report: Reviewer response for version 1

**DOI:** 10.21956/wellcomeopenres.25436.r109620

**Published:** 2024-11-15

**Authors:** Marcus C Stensmyr

**Affiliations:** 1 Lund University, Lund, Sweden

The manuscript presents the genome of *Tricholauxania praeusta, *a leaf fly (or lauxaniid fly). The genome has been sequenced as part of the Darwin Tree of Life (DTOL) project, and comprises 661MB assembled into 5 "chromosomes", as well as the mitochondrial genome. Annotation revealed 25,506 protein coding genes. The genome sequencing, assembly and annotation follows the standard DTOL state-of-the-art pipeline. The genome of a lauxaniid genome is a welcome addition to the field of Diptera genomics.

Are sufficient details of methods and materials provided to allow replication by others?

Yes

Is the rationale for creating the dataset(s) clearly described?

Yes

Are the datasets clearly presented in a useable and accessible format?

Yes

Are the protocols appropriate and is the work technically sound?

Yes

Reviewer Expertise:

Drosophila genomics and neurogenetics.

I confirm that I have read this submission and believe that I have an appropriate level of expertise to confirm that it is of an acceptable scientific standard.
